# Myocardial Infarction Caused by Aortic Root Thrombus in a Patient With Antiphospholipid Syndrome

**DOI:** 10.1002/ccr3.70256

**Published:** 2025-02-20

**Authors:** Kazuki Ichihara, Masataka Suzuki, Hiromi Hashimura, Hiroshi Eizawa

**Affiliations:** ^1^ Department of Cardiology Kobe City Nishi‐Kobe Medical Center Kobe Japan; ^2^ Department of Radiology Kobe University Graduate School of Medicine Kobe Japan

**Keywords:** antiphospholipid syndrome, aortic root thrombosis, cardiac computed tomography, cardiac magnetic resonance imaging, myocardial infarction

## Abstract

Aortic root thrombosis is a rare but serious complication of antiphospholipid syndrome that can lead to myocardial infarction. Cardiac computed tomography and magnetic resonance imaging are essential for noninvasively evaluating myocardial infarction and aortic root thrombosis.

## 1 Case Presentation

**FIGURE 1:**
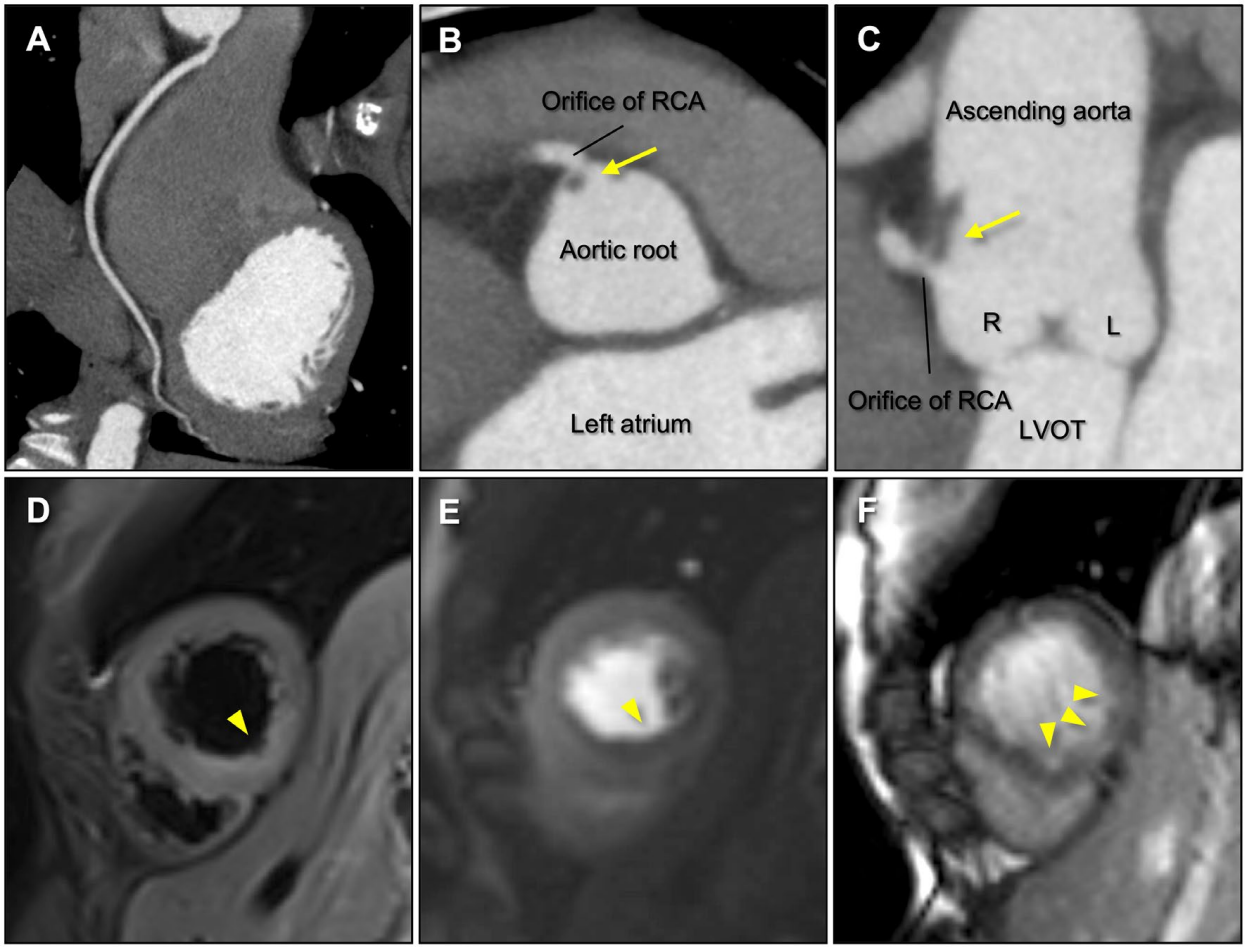
(A) On cardiac computed tomography, curved planar reconstruction image of the right coronary artery (RCA) shows no stenosis or obstruction. (B) Short‐axis image and (C) long‐axis image of the aortic root show a small thrombus near the RCA orifice (yellow arrows). (D) On cardiac magnetic resonance imaging, a high‐intensity area on the T2‐weighted short‐tau inversion recovery image indicates myocardial edema caused by acute myocardial infarction (yellow triangle). (E) Perfusion defect on the myocardial perfusion image indicates coronary flow restriction (yellow triangle). (F) Multiple subendocardial late gadolinium enhancements indicate subendocardial myocardial infarction (yellow triangles). L, left coronary aortic sinus; LVOT, left ventricular outflow tract; R, right coronary aortic sinus; RCA, right coronary artery.

A 26‐year‐old female with a history of systemic lupus erythematosus and antiphospholipid syndrome (APS) was admitted with chest pain. The patient had not been on regular medications, including aspirin, for 3 months. Electrocardiography showed mild T‐wave inversion in lead II without ST‐segment changes. Blood tests showed elevated high‐sensitivity cardiac troponin I (6908.7 pg/mL) and creatine kinase (328 IU/L). Transthoracic echocardiography revealed no left ventricular wall motion abnormalities or valvular diseases. Although acute coronary syndrome, coronary artery dissection, myocarditis, and pericarditis were suspected as the causes of chest pain and elevated cardiac enzymes, emergent invasive coronary angiography was withheld because the patient was hemodynamically stable. Cardiac computed tomography (CCT) and cardiac magnetic resonance imaging (CMR) were performed for further evaluation. CCT showed no significant stenosis or obstruction in the coronary arteries (Figure 1A). However, it revealed a small thrombus near the right coronary artery (RCA) orifice at the aortic root (Figure 1B,C). CMR showed a high‐intensity area on the T2‐weighted short‐tau inversion recovery image, a perfusion defect on the myocardial perfusion image, and multiple subendocardial late gadolinium enhancements in the inferior septum to the inferior wall (Figure 1D–F). These results indicated that the aortic root thrombus near the RCA orifice potentially caused a coronary flow restriction leading to subendocardial myocardial infarction (MI) or an embolic microvascular MI in the RCA territory. Invasive coronary angiography was not performed to avoid catheter‐induced systemic embolism. Following aspirin and warfarin administration, a repeat CCT showed the disappearance of the thrombus without any symptoms indicating systemic embolism, such as cerebral infarction.

## 2 Discussion

APS is an intractable autoimmune disease caused by antiphospholipid antibodies leading to arterial and/or venous thrombosis and pregnancy complications through various mechanisms, including direct endothelial damage, enhanced platelet aggregation, and endogenous anticoagulant inhibition. Cardiac manifestations of APS include MI, cardiomyopathy, heart valve lesions, and intracardiac thrombus. In particular, MI in APS tends to be caused by nonarteriosclerotic coronary thrombosis or embolism [1]. However, aortic thrombosis, especially in the aortic root, have been rarely reported because arterial thrombosis in APS mainly occurs in small‐ and medium‐sized vessels. Thrombus formation in APS requires secondary factors that promote complement activation, such as inflammation, infection, and trauma, in addition to an interaction of antiphospholipid antibodies with endothelial cells, platelets, neutrophils, and monocytes. The effects of previous aortitis complications or intimal injury might be associated with thrombus formation. Moreover, MI caused by aortic root thrombus has been previously reported, but it rarely causes MI without epicardial coronary artery obstruction, first detected by CMR [2]. A combination of low‐dose aspirin and warfarin is recommended to manage arterial thrombosis associated with APS, while direct oral anticoagulants are not recommended instead of warfarin [3].

In this patient, MI was considered to be caused by coronary flow restriction or microvascular embolism due to a small aortic root thrombus near the RCA orifice, diagnosed by CCT and CMR. Multimodal imaging assessment provides valuable information about the causes of MI in patients with APS.

## Author Contributions

**Kazuki Ichihara:** writing – original draft. **Masataka Suzuki:** writing – review and editing. **Hiromi Hashimura:** writing – review and editing. **Hiroshi Eizawa:** supervision.

## Consent

Written informed consent was obtained from the patient in line with the journal's patient consent policy for the publication of the case and accompanying images.

## Conflicts of Interest

The authors declare no conflicts of interest.

## Data Availability

Data available on request from the authors.
